# Unilateral Chewing Frequency and Self‐Reported Mastication Satisfaction: A Cross‐Sectional Study

**DOI:** 10.1111/joor.70212

**Published:** 2026-05-05

**Authors:** Carlo Ciotola, Mariam Hmeidan, Luca Contardo, Bachar Reda

**Affiliations:** ^1^ Department of Medical, Surgical and Health Sciences, School of Dentistry University of Trieste Trieste Italy; ^2^ Virtù Academy Tyre Lebanon; ^3^ Institut National de Santé Publique, d’Épidémiologie Clinique et de Toxicologie‐Liban (INSPECT‐LB) Beirut Lebanon

**Keywords:** depression, mastication satisfaction, oral behaviours, somatization, unilateral chewing behaviour

## Abstract

**Objective:**

This study aimed to assess the association between unilateral chewing frequency and self‐reported mastication satisfaction while taking into account other demographic and psychological factors.

**Methods:**

A cross‐sectional study was conducted between January 2018 and June 2020. Included participants were adult patients attending the Clinica di Chirurgia Maxillofacciale e Odontostomatologica di Trieste, Italy. Pearson chi‐square and independent samples t‐tests were used to determine the association between mastication satisfaction and unilateral chewing behaviour frequency, sex, depression and somatization scores. Afterwards, binary logistic regression was conducted to assess the relationship between self‐reported mastication satisfaction and unilateral chewing behaviours while controlling for covariates.

**Results:**

A total of 1425 participants with a mean age of 49.7 ± 17.6 years were included. Overall, 58.5% of the participants were females and the majority (83.3%) reported mastication satisfaction. Pearson chi‐square indicated a significant association between unilateral chewing frequency and mastication satisfaction (*p* < 0.001). Binary logistic regression indicated that frequent engagement in unilateral chewing behaviour was associated with a reduction in the odds of reporting satisfactory mastication. Study participants who reported unilateral chewing behaviour ‘all of the time’ showed the lowest likelihood of satisfaction (OR = 0.17). Female sex (OR = 0.69) and higher somatization scores (OR = 0.91) were associated with lower odds of mastication satisfaction, whereas higher depression scores were associated with slightly increased odds (OR = 1.04).

**Conclusion:**

Limited mastication satisfaction was associated with frequent unilateral chewing. These findings highlight the potential relevance of self‐regulation strategies in managing habitual chewing patterns and supporting masticatory performance.

## Graphical abstract

Frequent unilateral chewing is associated with reduced self‐reported mastication satisfaction. The lowest satisfaction was reported among individuals engaging in constant unilateral chewing, independent of demographic and psychological factors.

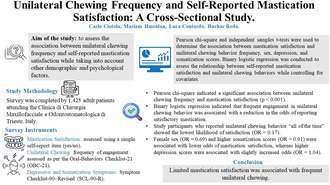

**Abbreviations**
DC/TMD: diagnostic criteria for temporomandibular disorders
EMA: ecological momentary assessment
EMI: ecological momentary intervention
FTUs: functional tooth units
MP: masticatory performance
OBC‐21: oral behaviours checklist‐21
OR: odds ratio
SCL‐90‐R: symptom checklist‐90‐revised
TMD: temporomandibular disorder

## 1 Introduction

The human body is a complex system influenced by a wide range of internal and external factors. Normal functional processes are influenced by physiological conditions, psychological stressors and habitual behaviours. Habitual behaviours are defined as automatic, persistent actions, often triggered by psychological conditions and performed with limited consideration of consequences [1, 2, 3]. Frequent engagement in such non‐goal directed behaviours, which are often triggered by situational cues and/or environmental stimuli, is known to affect the overall health‐related quality of life [4, 5].

To narrow the scope, in the field of dentistry, habitual oral behaviours are considered a subject of interest in both clinical and research settings. Assessment of oral behaviours is implemented within the patient record collection, such as the evaluation of Temporomandibular disorders (TMDs) [6]. Oral behaviours, defined as activities of the jaw muscles that are not related to eating or swallowing, have been reported to affect the overall functioning of the masticatory system. Frequent engagement in such behaviours has been linked to intensified TMD pain and jaw function limitations, according to various studies [7, 8, 9, 10, 11]. Moreover, engagement in such behaviours has been further linked to psychological triggers such as stress, anxiety and depression [12, 13, 14].

Assessment of such behaviours is usually carried out using self‐reported tools, more commonly the Oral Behaviours Checklist‐21, which is incorporated into the Diagnostic Criteria for TMD (DC/TMD) [6, 15, 16] and has been translated and validated in multiple languages [17, 18, 19, 20, 21]. Although oral behaviours are often evaluated collectively using total scores, item‐level analysis remains essential to help understand the role of each behaviour in the masticatory function and psychological outcomes. This approach is relevant considering the ongoing debates regarding the clinical relevance of certain OBC‐21 items, such as playing musical instruments [9] and findings suggesting that other behaviours, such as singing, lack a significant association with painful TMD conditions [22].

Among those behaviours, unilateral chewing (item‐16) has been identified as one of the prevalent oral behaviours in which individuals tend to chew on one side rather than engaging in bilateral mastication [11, 23, 24]. Such behaviour has been studied within the context of TMD being part of the OBC‐21 [25] and in relation to masticatory performance, with evidence indicating superior performance among bilateral chewers [26]. Additionally, a previous study has identified stress as a potential contributor for unilateral chewing behaviour [27]. However, the subjective consequences of adopting and engaging frequently in this habitual behaviour remain insufficiently studied. To date, no studies have examined the relationship between chewing side preference and self‐reported masticatory satisfaction.

Therefore, the aim of this study was to investigate the association between the frequency of unilateral chewing and self‐reported mastication satisfaction. Psychological factors, including depression and somatization, were included as covariates to account for their potential influence on both oral behaviours and perceived masticatory outcomes.

## 2 Materials and Methods

### 2.1 Study Design and Settings

A cross‐sectional study was conducted at the Clinica di Chirurgia Maxillofacciale e Odontostomatologica di Trieste, Italy, between January 2018 and June 2020. Included participants were all patients aged 18 years or above attending the dental clinic during the study period, requiring minimal and noninvasive dental services, such as routine oral examinations, supragingival scaling and polishing, application of topical fluoride and simple diagnostic radiographs. Prior to participation, a trained study representative explained the study objectives and data confidentiality procedures. Only individuals who provided written informed consent were enrolled. All procedures were in accordance with the ethical principles outlined in the Declaration of Helsinki by the World Medical Association for medical research involving human participants. Ethical approval for the study protocol was obtained from the Research and Ethics Committee (REC) of the Ateneo Units (IRB ID: n. 89/11062018).

### 2.2 Assessment Tool

Participants initially provided basic demographic information, including age and sex. Mastication satisfaction was assessed using a simple self‐report item (yes/no). The frequency of unilateral chewing during the preceding month was assessed using item 16 of the Italian version of the Oral Behaviours Checklist–21 (OBC‐21) [15]. Participants reported the recalled frequency of unilateral chewing using a five‐point Likert scale: none of the time, a little of the time, some of the time, most of the time or all of the time [6, 15, 28]. To control for potential psychological confounding, assessments of depressive symptoms and somatization were assessed using the Italian version of the Symptom Checklist‐90–Revised (SCL‐90‐R) [29, 30, 31, 32, 33, 34, 35]. The SCL‐90‐R contains 9 subscales: Depression, Somatization, Anxiety, Obsessive‐Compulsive, Interpersonal Sensitivity, Hostility, Phobic Anxiety, Paranoid Ideation and Psychoticism [32, 36]. For this study, participants were required to respond to a series of items belonging to the depression and somatization subscales. Answers were reported using a five‐point scale ranging from 0 (not at all) to 4 (extremely), with higher total scores indicating greater symptom severity [36]. The depression subscale consisted of 13 items, resulting in a total score range of 0–52, while the somatization subscale included 12 items with a total score range of 0–48.

### 2.3 Sample Size Calculation

A single population proportion formula was used to calculate the minimal sample size needed for this study due to the lack of comparable studies among the Italian population. Epi Info version 7 was used while assuming a conservative prevalence estimate of 50%, a 95% confidence interval and a margin of error of 5%. The minimum sample required was 384 participants, which was further expanded to 1425 participants for the purpose of increasing the statistical power and enhancing the generalizability of the findings.

$$N=\frac{{\mathrm{Z\alpha}}^{2}\times P\;\left(1-P\right)}{d^{2}}=\frac{1.96^{2}\times 0.05\left(1-0.05\right)}{0.05^{2}}=384$$

### 2.4 Statistical Analysis

Data were analysed using SPSS (version 26, IBM Corp., Armonk, NY, USA) and a *p* < 0.05 was considered statistically significant. To summarize the characteristics of the study participants, descriptive statistics were employed using mean ± standard deviation for continuous variables, while categorical variables were reported in means of percentages and frequencies. Cross‐tabulation was performed to describe the distribution of mastication satisfaction across the categories of unilateral chewing behaviour. Afterwards, bivariate analyses were conducted to examine associations between mastication satisfaction and study variables using the chi‐square test for categorical variables and the independent samples *t*‐test for continuous variables. Binary logistic regression analysis was then performed to evaluate the association between unilateral chewing frequency and mastication satisfaction while adjusting for age, sex, depression and somatization. Model assumptions were assessed prior to analysis, including evaluation of multicollinearity; all correlation coefficients were below 0.8, indicating no significant multicollinearity.

## 3 Results

A total of 1425 participants were included in the analysis. The mean age was 49.7 ± 17.6 years, of which 58.5% (*n* = 833) were females and 41.5% (*n* = 592) were males. Mean psychological assessment scores, as measured by the SCL‐90‐R, indicated generally low levels of depressive and somatization symptoms, with mean scores of 7.5 ± 7.0 for depression and 7.0 ± 7.8 for somatization.

Overall, the majority of the study population reported satisfied mastication (*n* = 1187, 83.3%), while 16.7% (*n* = 238) reported limited mastication satisfaction. Regarding the unilateral chewing behaviour, 36.5% reported no engagement, 27.9% reported engagement ‘a little of the time’ 17.6% ‘some of the time’ 14% ‘most of the time’ and 3.9% ‘all of the time’. Among participants who did not engage in unilateral chewing, 91.2% reported satisfactory mastication and 8.8% reported limited satisfaction. The proportion of participants reporting limited mastication satisfaction increased progressively across higher unilateral chewing frequency categories, reaching 50% among those who reported engaging in unilateral chewing ‘all of the time’. Detailed distributions are presented in Table 1.

**TABLE 1:** Distribution of mastication satisfaction across levels of unilateral chewing frequency.

Mastication Satisfaction	Unilateral Chewing Frequency				
	None of the time	A little of the Time	Some of the time	Most of the time	All of the time
	*n* (%)	*n* (%)	*n* (%)	*n* (%)	*n* (%)
Not Satisfied	46 (8.8%)	63 (15.8%)	50 (19.9%)	51 (25.5%)	28 (50.0%)
Satisfied	474 (91.2%)	335 (84.2%)	201 (80.1%)	149 (74.5%)	28 (50.0%)
Total	520 (100%)	398 (100%)	251 (100%)	200 (100%)	56 (100%)

Bivariate analyses indicated significant associations between self‐reported mastication satisfaction and all examined variables. Pearson chi‐square tests showed significant associations between mastication satisfaction and unilateral chewing frequency (*p* < 0.001) as well as sex (*p* < 0.001). Independent samples *t*‐tests indicated significant differences in mean depression scores (*p* < 0.001) and somatization scores (*p* < 0.001) according to mastication satisfaction status.

Binary logistic regression was performed to assess the association between self‐reported mastication satisfaction and unilateral chewing frequency while adjusting for age, sex, depression and somatization scores as relevant covariates. The full regression results, including regression coefficient (β), odds ratios (ORs), 95% confidence intervals (CIs) and *p*‐values are presented in Table 2. Compared with participants who did not engage in unilateral chewing, the odds of reporting satisfactory mastication decreased progressively with increasing frequency of unilateral chewing. Engagement in unilateral chewing behaviour ‘a little of the time’ was associated with a 42.6% lower odds of reporting mastication satisfaction compared to those who did not engage in the behaviour (β = −0.555 or = 0.574). Similarly, engagement in this behaviour ‘some of the time’ was associated with 47.2% lower odds of mastication satisfaction (β = −0.638 or = 0.528) and ‘most of the time’ with 57.9% lower odds (β = −0.865 or = 0.421). Participants who reported unilateral chewing ‘all of the time’ had the lowest odds of reporting satisfactory mastication, with an 82.6% reduction compared to those who did not report engagement in the behaviour (β = −1.747 or = 0.174). Among covariates, females had 31.3% lower odds of reporting satisfactory mastication compared to males (β = −0.375 or = 0.687). Higher depression scores were associated with a modest increase in the odds of mastication satisfaction by 3.6% (β = 0.036 or = 1.036), whereas higher somatization scores were associated with reduced odds of mastication satisfaction by 9.2% (β = −0.097 or = 0.908).

**TABLE 2:** Predictors of Mastication Satisfaction (Yes/No).

Variable	B	*p*	Odds ratio (OR)	95% CI for OR	
				Lower	Upper
Age	0.007	0.122	1.007	0.998	1.016
Sex (Female)	−0.375	0.021*	0.687	0.500	0.944
A little of time unilateral chewing frequency.	−0.555	0.008*	0.574	0.380	0.867
Some of the time unilateral chewing frequency.	−0.638	0.006*	0.528	0.336	0.832
Most of the time unilateral chewing frequency.	−0.865	< 0.001*	0.421	0.265	0.669
All of the time unilateral chewing frequency.	−1.747	< 0.001*	0.174	0.091	0.336
Total Depression Score.	0.036	0.011*	1.036	1.008	1.065
Total Somatization Score.	−0.097	< 0.001*	0.908	0.880	0.936

* *Note: p* < 0.05 indicates statistical significance.

## 4 Discussion

This study investigated the association between unilateral chewing behaviour frequency and self‐reported mastication satisfaction while controlling for demographic and psychological factors. Findings revealed the potential association between the degree of engagement in such behaviour and limited masticatory satisfaction.

While masticatory performance (MP), defined as the ability of the oral apparatus to crush and grind food over a predetermined number of mastication cycles [37, 38], is commonly used for objective assessment of mastication and has been assessed in previous studies in relation to unilateral versus bilateral chewing patterns [26, 39, 40], subjective assessment of masticatory satisfaction and unilateral chewing behaviour is also important in providing complementary insight into individuals’ perceived functional outcomes. Previous research indicated that the self‐reported chewing side often does not align with objective measures and may reflect functional asymmetries rather than conscious perception of mastication [26, 41, 42]. While subjective masticatory satisfaction has not been systematically assessed, the present study addresses this gap by examining the relationship between unilateral chewing frequency and individuals’ perceived masticatory satisfaction, providing novel insight into subjective awareness of functional oral outcomes.

Bivariate analysis revealed a significant association between the frequency of engagement in unilateral behaviour and mastication satisfaction. When controlling for potential confounding factors, the association remained significant, indicating that individuals who engage more frequently in the unilateral chewing behaviour reported lower mastication satisfaction compared with bilateral chewers. These findings suggest that the impact of oral behaviours is not only detectable through objective clinical assessment but is also subjectively perceived by individuals.

From a behavioural perspective, engagement in habitual behaviours requires minimal cognitive effort [43, 44, 45], resulting in an automatic engagement with no regard to the potential outcomes. Disrupting engagement in such behaviours, especially those with negative outcomes for the individuals, generally requires an initial increase in awareness [46]. When the individual becomes aware of the negative impact of engagement in harmful behaviours, this knowledge represents a critical step toward inhibiting the chain of unconscious engagement in this behaviour and hence self‐regulation can be implemented [47]. Self‐regulation has been identified to pose direct effects on healthy habits and mental health, leading to an indirect positive impact on well‐being [48].

As for the other variables, females reported limited masticatory satisfaction compared to their male counterparts. This finding can be explained by the sex‐related difference in masticatory performance reported in previous studies, where males showed higher maximal occlusal bite force [49, 50]. Additionally, somatization was found to be associated with reduced mastication satisfaction, in parallel with previous studies suggesting that patients experiencing orofacial pain reported difficulties in eating and reduced life satisfaction compared to pain‐free individuals [51, 52]. In contrast, depression showed a positive association with masticatory satisfaction. Although previous studies indicated reduced subjective and objective mastication ability in individuals with depression, those results were shown in patients with higher depression scores [53, 54]. This discrepancy may be explained by the relatively low depression scores in our sample, the limited sensitivity of the binary ‘Yes/No’ mastication satisfaction measure and the generally healthy dental clinic population requiring minimal interventions. Nevertheless, inclusion of depression as a covariate was essential to control for its potential confounding effect and the finding should be interpreted with caution.

This study was the first to assess the potential association between self‐reported mastication satisfaction and unilateral chewing behaviour, a prevalent behaviour with a known impact on overall oral health function. The sample size used was beyond the minimal sample size needed for the purpose of enhancing the power and generalizability of the findings. Additionally, regression analysis was conducted while taking into account potential confounding factors, thus strengthening the robustness of the results. Findings from this study indicated that individuals who frequently engaged in unilateral chewing behaviour were capable of reporting limited masticatory satisfaction. Clinically, this awareness may represent an initial step toward behaviour modification. Clinician‐guided identification of harmful oral habits, combined with education and self‐regulation strategies, may help disrupt automatic engagement in unilateral chewing and promote bilateral and mindful eating behaviours. Such approaches align with Ecological Momentary Assessment and Intervention (EMA/EMI) principles, which emphasize real‐time identification and modification of behaviours within daily contexts [55, 56, 57]. Longitudinal studies assessing the impact of self‐regulation strategies in modifying habitual oral behaviours are important to identify the impact of conscious triggering on the engagement in habitual behaviours. This study contributes to Sustainable Development Goal number three (SDG 3): Good Health and Well‐being, one of the goals of the United Nations 2030 Agenda for Sustainable Development, by highlighting the relationship between mastication satisfaction and habitual unilateral chewing behaviour, thus emphasizing the importance of adopting bilateral and mindful eating habits [58].

On the other hand, several limitations shall be reported. First, the cross‐sectional design limits the ability to establish causality. Second, participants were recruited from dental clinic attendees requiring only minimal, noninvasive care to reduce inclusion of severe dental conditions that could confound self‐perceived mastication. However, participation rates and reasons for non‐participation were not systematically recorded, limiting formal assessment of selection bias. Although the final sample was large (*n* = 1425), well above the minimum required sample (*n* = 384), potential participation‐related influencing factors cannot be excluded. Third, the study relied on self‐reported measures, which may result in information bias, including recall and reporting bias. In particular, mastication satisfaction was assessed using a single dichotomous item, reflecting perceived experience rather than objective function. Similarly, chewing‐side preference was measured via a self‐reported OBC‐21 item, a widely used tool in clinical and research settings. Although several validated objective and subjective methods exist for assessing masticatory performance and chewing‐side preference, such as the chewing gum test (CG), asymmetry index (ASI) and the visual analog scale (VAS) [38, 59, 60], these were not employed in the present study. The primary aim was to evaluate individuals’ self‐perceived masticatory experience and awareness of behavioural consequences rather than objective functional capacity. Importantly, Functional Tooth Units (FTUs) or occluding pairs, primary determinants of masticatory satisfaction, were not collected. Consequently, it cannot be determined whether unilateral chewing reflected functional necessity or habitual behaviour. Additional clinical variables, including TMD, malocclusion, arch integrity, teeth alignment oral health status and use of dental prostheses, as well as sociodemographic characteristics, were not assessed, further restricting the scope of conclusions. Finally, as with most observational studies, the findings may be susceptible to systemic bias, including potential selection bias, information bias and unmeasured confounding, which could influence the observed associations [61]. Future studies are recommended to address these limitations.

## 5 Conclusion

The findings from this study identified the presence of a significant association between limited masticatory satisfaction and frequent engagement in unilateral chewing behaviour. Self‐regulating strategies, including clinician‐guided awareness and self‐monitoring of harmful oral behaviours, may help individuals adopt bilateral and mindful eating strategies, potentially supporting masticatory performance. Further longitudinal and interventional studies are warranted to confirm these implications.

## Author Contributions

Concept and design aspects were led by M.H. and B.R. Data acquisition and statistical analysis were conducted by C.C, M.H. and B.R. Analysis and interpretation of the data were performed collectively by C.C, M.H., L.C. and B.R. M.H. was responsible for drafting the manuscript, which was critically revised for important intellectual content by L.C. and B.R. All authors reviewed and approved the final manuscript.

## Funding

The authors have nothing to report.

## Ethics Statement

Prior to participation, a trained study representative explained the study objectives and data confidentiality procedures. Only individuals who provided written informed consent were enrolled. All procedures were in accordance with the ethical principles outlined in the Declaration of Helsinki by the World Medical Association for medical research involving human participants. Ethical approval for the study protocol was obtained from the Research and Ethics Committee (REC) of the Ateneo Units (IRB ID: n. 89/11062018).

## Conflicts of Interest

The authors declare no conflicts of interest.

## Data Availability

The data that support the findings of this study are available from the corresponding author upon reasonable request.

## References

[1] R. M. Hardwick , A. D. Forrence , J. W. Krakauer , and A. M. Haith , “Time‐Dependent Competition Between Goal‐Directed and Habitual Response Preparation,” Nature Human Behaviour 3 (2019): 1252–1262. 10.1038/s41562-019-0725-0 31570762

[2] S. de Wit , P. Watson , H. A. Harsay , M. X. Cohen , I. van de Vijver , and K. R. Ridderinkhof , “Corticostriatal Connectivity Underlies Individual Differences in the Balance Between Habitual and Goal‐Directed Action Control,” Journal of Neuroscience 32 (2012): 12066–12075. 22933790 10.1523/JNEUROSCI.1088-12.2012 PMC6621537

[3] S. N. Handel and R. J. Smith , “Making and Breaking Habits: Revisiting the Definitions and Behavioural Factors That Influence Habits in Animals,” Journal of the Experimental Analysis of Behaviour 121 (2024): 8–26. 10.1002/jeab.889 PMC10842199 38010353

[4] F. G. Ashby , B. O. Turner , and J. C. Horvitz , “Cortical and Basal Ganglia Contributions to Habit Learning and Automaticity,” Trends in Cognitive Sciences 14 (2010): 208–215. 20207189 10.1016/j.tics.2010.02.001 PMC2862890

[5] J. De Houwer , “On How Definitions of Habits Can Complicate Habit Research,” Frontiers in Psychology 10 (2019): 2642. 31849762 10.3389/fpsyg.2019.02642 PMC6895142

[6] E. Schiffman , R. Ohrbach , E. Truelove , International RDC/TMD Consortium Network, International association for Dental Research , Orofacial Pain Special Interest Group, International Association for the Study of Pain , et al., “Diagnostic Criteria for Temporomandibular Disorders (DC/TMD) for Clinical and Research Applications: Recommendations of the International RDC/TMD Consortium Network* and Orofacial Pain Special Interest Group†,” Journal of Oral & Facial Pain and Headache 28 (2014): 6–27. 24482784 10.11607/jop.1151 PMC4478082

[7] B. Reda , F. Lobbezoo , L. Contardo , et al., “TMD Patients With High Pain‐Related Disability Have Higher Oral Behaviour Scores Than Individuals Without Pain and TMD Patients With Low Pain‐Related Disability,” Journal of Oral Rehabilitation 52 (2025): 2059–2065. 40634242 10.1111/joor.70013

[8] C. Barbosa , M. C. Manso , T. Reis , T. Soares , S. Gavinha , and R. Ohrbach , “Are Oral Overuse Behaviours Associated With Painful Temporomandibular Disorders? A Cross‐Sectional Study in Portuguese University Students,” Journal of Oral Rehabilitation 48 (2021): 1099–1108. 34273189 10.1111/joor.13226

[9] V. Donnarumma , R. Ohrbach , V. Simeon , F. Lobbezoo , N. Piscicelli , and A. Michelotti , “Association Between Waking‐State Oral Behaviours, According to the Oral Behaviours Checklist and TMD Subgroups,” Journal of Oral Rehabilitation 48 (2021): 996–1003. 34192368 10.1111/joor.13221 PMC8457156

[10] W. Keela , T. Itthikul , S. Mitrirattanakul , and S. Pongrojpaw , “Awake and Sleep Oral Behaviours in Patients With Painful Temporomandibular Disorders,” International Dental Journal 74 (2024): 138–145. 37586995 10.1016/j.identj.2023.07.013 PMC10829361

[11] L. Xu , B. Cai , S. Fan , S. Lu , and K. Dai , “Association of Oral Behaviours With Anxiety, Depression and Jaw Function in Patients With Temporomandibular Disorders in China: A Cross‐Sectional Study,” Medical Science Monitor 27 (2021): e929985. 33999914 10.12659/MSM.929985 PMC8139132

[12] B. Reda , F. Lobbezoo , L. Contardo , G. Zanon , G. Aarab , and D. Manfredini , “Association Between Oral Behaviours and Generalized Anxiety in a Sample of University Students: A Cross‐Sectional Study,” Cranio (2025): 1–9. 10.1080/08869634.2025.2591552 41287391

[13] O. I. Saracutu , D. Manfredini , A. Bracci , E. Ferrari Cagidiaco , M. Ferrari , and A. Colonna , “Self‐Reported Mandible Bracing and Teeth Clenching Are Associated With Anxiety and Depression Traits in a Group of Healthy Young Individuals,” Journal of Oral & Facial Pain and Headache 38 (2024): 85–90. 39800959 10.22514/jofph.2024.041 PMC11810672

[14] Y. Zhong , F. Luo , X. Li , et al., “Associations Between Oral Behaviours, Temporomandibular Disorder Subtypes and Psychological Distress in Adult Women: A Retrospective Case‐Control Study,” Journal of Oral & Facial Pain and Headache 38 (2024): 87–99. 39800575 10.22514/jofph.2024.030 PMC11810668

[15] M. R. Markiewicz , R. Ohrbach , and W. D. McCall , “Oral Behaviours Checklist: Reliability of Performance in Targeted Waking‐State Behaviours,” Journal of Orofacial Pain 20 (2006): 306–316. 17190029

[16] R. Ohrbach , “Psychometric Properties of the Oral Behaviours Checklist: Preliminary Findings,” Journal of Dental Research 83 (2004): 1194.

[17] C. Barbosa , M. C. Manso , T. Reis , T. Soares , S. Gavinha , and R. Ohrbach , “Cultural Equivalence, Reliability and Utility of the Portuguese Version of the Oral Behaviours Checklist,” Journal of Oral Rehabilitation 45 (2018): 924–931. 30187498 10.1111/joor.12716

[18] V. Donnarumma , I. Cioffi , A. Michelotti , R. Cimino , S. Vollaro , and M. Amato , “Analysis of the Reliability of the Italian Version of the Oral Behaviours Checklist and the Relationship Between Oral Behaviours and Trait Anxiety in Healthy Individuals,” Journal of Oral Rehabilitation 45 (2018): 317–322. 29420851 10.1111/joor.12614

[19] F. N. Tedin Ng , K. Kadir , and Z. Y. M. Yusof , “Reliability and Validity of the Malaysian English Version of the Diagnostic Criteria for Temporomandibular Disorder (M‐English DC/TMD),” Healthcare (Basel) 10 (2022): 329. 35206943 10.3390/healthcare10020329 PMC8871999

[20] M. J. van der Meulen , F. Lobbezoo , I. H. A. Aartman , and M. Naeije , “Validity of the Oral Behaviours Checklist: Correlations Between OBC Scores and Intensity of Facial Pain,” Journal of Oral Rehabilitation 41 (2014): 115–121. 24274580 10.1111/joor.12114

[21] B. Reda , F. Lobbezoo , A. El‐Outa , et al., “Translation and Cultural Adaptation of an Arabic Version of the OBC‐21,” Cranio 44 (2025): 1–7. 40839342 10.1080/08869634.2025.2548436

[22] M. K. A. van Selms , B. Reda , C. M. Visscher , D. Manfredini , and F. Lobbezoo , “The Effect of Singing on Pain and Psychological Well‐Being in a Patient Population With Pain‐Related Temporomandibular Disorders,” Journal of Oral Rehabilitation 49 (2022): 841–848. 35570643 10.1111/joor.13339

[23] B. Reda , F. Lobbezoo , L. Contardo , et al., “Prevalence of Oral Behaviours in General Dental Patients Attending a University Clinic in Italy,” Journal of Oral Rehabilitation 50 (2023): 370–375. 36718600 10.1111/joor.13427

[24] J. Han , S.‐H. Choi , H. J. Ahn , J. S. Kwon , Y. Park , and Y. J. Choi , “Unilateral Temporomandibular Joint Disorders Diagnosed as Both Disc Displacement Without Reduction and Osteoarthritis Show Vertical Craniofacial Asymmetry in Women,” Journal of Oral & Facial Pain and Headache 38 (2024): 77–86. 10.22514/jofph.2024.029 PMC11810645 39800574

[25] L. Cardoso‐Silva , B. C. Gomes , R. P. de Faria Melo , et al., “Unilateral Molar Incisor Hypomineralization Influences the Chewing Side? An Observational Study in Children,” Clinical Oral Investigations 28 (2024): 634. 39511031 10.1007/s00784-024-06037-y

[26] S. G. Farias Gomes , W. Custodio , J. S. Moura Jufer , A. A. del Bel Cury , and R. C. M. Rodrigues Garcia , “Correlation of Mastication and Masticatory Movements and Effect of Chewing Side Preference,” Brazilian Dental Journal 21 (2010): 351–355. 20976387 10.1590/s0103-64402010000400011

[27] S. Bano , P. Jagan , S. S. Kumar , H. Shalma , D. Swathi , and A. Arun Prasath , “Association Between Unilateral Chewing Habit and Mental Stress and Anxiety Among Young Adults ‐ A Cross‐Sectional Study,” Clinical Dentistry (0974–3979) 18 (2024): 18.

[28] R. Ohrbach , Diagnostic Criteria for Temporomandibular Disorders: Assessment Instruments (2014).

[29] L. R. Derogatis and P. A. Cleary , “Confirmation of the Dimensional Structure of the Scl‐90: A Study in Construct Validation,” Journal of Clinical Psychology 33 (1977): 981–989.

[30] M. A. Vallejo , C. M. Jordán , M. I. Díaz , M. I. Comeche , and J. Ortega , “Psychological Assessment via the Internet: A Reliability and Validity Study of Online (Vs Paper‐And‐Pencil) Versions of the General Health Questionnaire‐28 (GHQ‐28) and the Symptoms Check‐List‐90‐Revised (SCL‐90‐R),” Journal of Medical Internet Research 9 (2007): e2. 17478411 10.2196/jmir.9.1.e2 PMC1794673

[31] A. Prunas , I. Sarno , E. Preti , F. Madeddu , and M. Perugini , “Psychometric Properties of the Italian Version of the SCL‐90‐R: A Study on a Large Community Sample,” European Psychiatry 27 (2012): 591–597. 21334861 10.1016/j.eurpsy.2010.12.006

[32] L. R. Derogatis and R. Unger , “Symptom Checklist‐90‐Revised,” in The Corsini Encyclopedia of Psychology, ed. I. B. Weiner and W. E. Craighead (Wiley, 2010), 1–2.

[33] R. Gomez , L. Karimi , K. Hein , S. Papapetrou , and V. Stavropoulos , “Symptom Checklist‐90‐Revised: Factor Structure and Relevance as a Measure for the Hierarchical Taxonomy of Psychopathology Model,” International Journal of Mental Health 55 (2024): 1–24.

[34] L. R. Derogatis , K. Rickels , and A. F. Rock , “The SCL‐90 and the MMPI: A Step in the Validation of a New Self‐Report Scale,” British Journal of Psychiatry 128 (1976): 280–289. 10.1192/bjp.128.3.280 1252693

[35] U. Prinz , D. O. Nutzinger , H. Schulz , F. Petermann , C. Braukhaus , and S. Andreas , “Comparative Psychometric Analyses of the SCL‐90‐R and Its Short Versions in Patients With Affective Disorders,” BMC Psychiatry 13 (2013): 104. 23537095 10.1186/1471-244X-13-104 PMC3626675

[36] Y. Yu , C. Wan , X. Zhao , et al., “Undergraduate Students’ Norms for the Chinese Version of the Symptom Check‐List‐90‐R (SCL‐90‐R),” BMC Public Health 20 (2020): 1588. 33087089 10.1186/s12889-020-09689-z PMC7579932

[37] J. F. Bates , G. D. Stafford , and A. Harrison , “Masticatory Function ‐ a Review of the Literature. III. Masticatory Performance and Efficiency,” Journal of Oral Rehabilitation 3 (1976): 57–67. 772184 10.1111/j.1365-2842.1976.tb00929.x

[38] T. M. S. V. Gonçalves , M. Schimmel , A. van der Bilt , et al., “Consensus on the Terminologies and Methodologies for Masticatory Assessment,” Journal of Oral Rehabilitation 48 (2021): 745–761. 33638156 10.1111/joor.13161 PMC8252777

[39] m. y. Yun , J.‐H. Jang , and C. GaeSig , “Comparison of Masticatory Performance and Efficiency Between Bilateral and Unilateral Chewing in Adults,” Journal of Transdisciplinary Studies 9 (2025): 97–103.

[40] B. Reda , M. Hmeidan , M. Masuino , and L. Contardo , “Evaluation of Objective Masticatory Performance: An Experimental Study in Healthy Adults,” Saudi Dent J 37 (2025): 67. 41118003 10.1007/s44445-025-00022-1 PMC12540237

[41] J. Martinez‐Gomis , M. Lujan‐Climent , S. Palau , J. Bizar , J. Salsench , and M. Peraire , “Relationship Between Chewing Side Preference and Handedness and Lateral Asymmetry of Peripheral Factors,” Archives of Oral Biology 54 (2009): 101–107. 18947820 10.1016/j.archoralbio.2008.09.006

[42] B. Rovira‐Lastra , E. I. Flores‐Orozco , J. Salsench , M. Peraire , and J. Martinez‐Gomis , “Is the Side With the Best Masticatory Performance Selected for Chewing?,” Archives of Oral Biology 59 (2014): 1316–1320. 25173664 10.1016/j.archoralbio.2014.08.005

[43] P. Lally and B. Gardner , “Promoting Habit Formation,” Health Psychology Review 7 (2013): S137–S158.

[44] W. Wood and D. Rünger , “Psychology of Habit,” Annual Review of Psychology 67 (2016): 289–314. 10.1146/annurev-psych-122414-033417 26361052

[45] A. G. Harvey , C. A. Callaway , G. G. Zieve , N. B. Gumport , and C. C. Armstrong , “Applying the Science of Habit Formation to Evidence‐Based Psychological Treatments for Mental Illness,” Perspectives on Psychological Science 17 (2022): 572–589. 34495781 10.1177/1745691621995752 PMC12318445

[46] J. Sánchez‐Cañizares , “The Role of Consciousness in Triggering Intellectual Habits,” Frontiers in Human Neuroscience 8 (2014): 312. 24904355 10.3389/fnhum.2014.00312 PMC4033233

[47] C. A. Gianessi , “From Habits to Self‐Regulation: How Do We Change?,” Yale Journal of Biology and Medicine 85 (2012): 293–299. 22737058 PMC3375665

[48] S. S. Sousa , M. M. Ferreira , S. Cruz , A. Sampaio , and A. Silva‐Fernandes , “A Structural Equation Model of Self‐Regulation and Healthy Habits as an Individual Protective Tool in the Context of Epidemics–Evidence From COVID‐19,” Frontiers in Psychology 12 (2021): 696813. 34594265 10.3389/fpsyg.2021.696813 PMC8476840

[49] M. Sano and H. Shiga , “Gender Differences in Masticatory Function in Elderly Adults With Natural Dentition,” Odontology 109 (2021): 973–978. 34228214 10.1007/s10266-021-00622-3

[50] H. Shiga , Y. Kobayashi , H. Katsuyama , M. Yokoyama , and I. Arakawa , “Gender Difference in Masticatory Performance in Dentate Adults,” Journal of Prosthodontic Research 56 (2012): 166–169. 22613955 10.1016/j.jpor.2012.02.001

[51] T.‐C. Chen and C.‐S. Lin , “Complex Associations Between Pain and Masticatory Function in Patients With Muscular Temporomandibular Disorders,” Journal of Oral Rehabilitation 52 (2025): 2084–2094. 40685685 10.1111/joor.14052

[52] I. A. Boggero , M. V. Rojas‐Ramirez , R. de Leeuw , and C. R. Carlson , “Satisfaction With Life in Orofacial Pain Disorders: Associations and Theoretical Implications,” Journal of Oral & Facial Pain and Headache 30 (2016): 99–106. 27128473 10.11607/ofph.1526 PMC4983167

[53] H. Roohafza , H. Afshar , A. H. Keshteli , et al., “Masticatory Ability With Depression, Anxiety and Stress: Does There Exist Any Association?,” Dent Res J (Isfahan) 13 (2016): 211–216. 27274340 10.4103/1735-3327.182179 PMC4878204

[54] X. Wei , X. Zhang , X. Zhang , et al., “Association Between Masticatory Function and Depression in Older Adults: Results From NHANES 2009 to 2018,” Journal of Clinical Periodontology 51 (2024): 1458–1465. 38987924 10.1111/jcpe.14046

[55] S. Shiffman , A. A. Stone , and M. R. Hufford , “Ecological Momentary Assessment,” Annual Review of Clinical Psychology 4 (2008): 1–32. 10.1146/annurev.clinpsy.3.022806.091415 18509902

[56] M. R. Postma , S. Vrancken , M. Daemen , et al., “Working Mechanisms of the Use and Acceptability of Ecological Momentary Interventions: A Realist Evaluation of a Guided Self‐Help Ecological Momentary Intervention Targeting Self‐Esteem,” BMC Public Health 24 (2024): 1633. 38898412 10.1186/s12889-024-19143-z PMC11186172

[57] M. E. McDevitt‐Murphy , M. T. Luciano , and R. J. Zakarian , “Use of Ecological Momentary Assessment and Intervention in Treatment With Adults,” Focus (Am Psychiatr Publ) 16 (2018): 370–375. 31191181 10.1176/appi.focus.20180017 PMC6493252

[58] B. X. Lee , F. Kjaerulf , S. Turner , et al., “Transforming Our World: Implementing the 2030 Agenda Through Sustainable Development Goal Indicators,” Journal of Public Health Policy 37, no. 1 (2016): 13–31. 27638240 10.1057/s41271-016-0002-7

[59] Q. Olivieri , S. Maniewicz , N. Chebib , P. Mojon , and F. Müller , “Three Tests to Determine a Preferred Chewing Side in Partially Edentulous Patients: A Pilot Study,” Journal of Prosthetic Dentistry 133 (2025): 163–168. 37802735 10.1016/j.prosdent.2023.09.003

[60] E. I. Flores‐Orozco , B. Rovira‐Lastra , M. Peraire , J. Salsench , and J. Martinez‐Gomis , “Reliability of a Visual Analog Scale for Determining the Preferred Mastication Side,” Journal of Prosthetic Dentistry 115 (2016): 203–208. 26455536 10.1016/j.prosdent.2015.07.006

[61] J. C. Bond , M. P. Fox , L. A. Wise , and B. Heaton , “Quantitative Assessment of Systematic Bias: A Guide for Researchers,” Journal of Dental Research 102 (2023): 1288–1292. 37786916 10.1177/00220345231193314 PMC10714380

